# Quality of Life of Colorectal Cancer Patients Treated with Chemotherapy

**DOI:** 10.3390/nu18020191

**Published:** 2026-01-07

**Authors:** Monika Ziętarska, Sylwia Małgorzewicz

**Affiliations:** 1 Clinic of Pediatrics, Hematology and Oncology, University Clinical Center in Gdańsk, Dębinki 7, 80-952 Gdansk, Poland; 2 Department of Clinical Nutrition, Medical University of Gdańsk, Dębinki 7, 80-211 Gdansk, Poland; sylwia.malgorzewicz@gumed.edu.pl

**Keywords:** colorectal cancer, oral nutritional supplements, health-related quality of life, pre-cachexia, functional status

## Abstract

**Background/Objectives:** Colorectal cancer (CRC) is associated with anorexia–cachexia syndrome, which negatively affects health-related quality of life (HRQoL). This study aimed to evaluate HRQoL and functional status in CRC patients undergoing chemotherapy who were eligible for oral nutritional supplementation (ONS). **Methods:** In this prospective, randomized study, 72 patients with stage II–IV CRC were enrolled (40 intervention group [IG], 32 control group [CG]). IG received ONS (2 × 125 mL/day, 600 kcal, 36 g protein) for 12 weeks, while CG received dietary counseling only. HRQoL was assessed every 4 weeks with the Functional Assessment of Anorexia/Cachexia Therapy (FAACT, version 4.0). Functional status was evaluated with the Karnofsky scale. Nutritional status was assessed using the Subjective Global Assessment (SGA), Nutritional Risk Screening (NRS-2002), and body mass index (BMI), and appetite was assessed on a visual analogue scale (VAS). Clinical Trial Registration: ClinicalTrials.gov, NCT02848807. **Results:** Mean FAACT score did not differ significantly between groups over 12 weeks (101.0 ± 22.8, 95% CI: 94.6–107.4 vs. 105.1 ± 21.4, 95% CI: 99.1–111.1; *p* = 0.06). However, the observed difference corresponded to an effect size at the lower bound of the moderate range. However, minimally important difference (MID) analysis demonstrated that clinically meaningful improvement was significantly more frequent in IG than in CG for global FAACT (32% vs. 8%; *p* = 0.03, OR = 5.50, 95% CI: 1.10–27.62, φ = 0.29), physical well-being (32% vs. 8%; *p* = 0.03, OR = 5.50, 95% CI: 1.10–27.62, φ = 0.29), and emotional well-being (38% vs. 4%; *p* = 0.002, OR = 14.86, 95% CI: 1.79–123.36, φ = 0.40). Functional well-being and anorexia/cachexia concerns showed favorable, but nonsignificant, trends (FWB improvement: 29% vs. 8%, *p* = 0.05, OR = 4.79, 95% CI: 0.95–24.27, φ = 0.26; ACS deterioration: 3% vs. 20%, *p* = 0.07, OR = 0.12, 95% CI: 0.01–1.11, φ = 0.28). HRQoL correlated positively with nutritional status, appetite, and functional performance, while Karnofsky scores remained stable in both groups. **Conclusions:** ONS did not significantly change the mean QoL scores at the group level but increased the proportion of patients achieving clinically meaningful improvement, particularly in the physical and emotional domains. These findings suggest that ONS may benefit selected patients who respond to nutritional interventions, underscoring the clinical relevance of individualized nutrition strategies in oncology.

## 1. Introduction

Colorectal cancer represents a significant public health problem. It is the third most commonly diagnosed malignancy and the second leading cause of cancer-related death worldwide. In 2020, 1.9 million new cases of CRC were reported, along with 930,000 deaths. Researchers project that by 2040, the incidence of new cases will increase to 3.2 million, while mortality will rise to 1.6 million [1,2].

A diagnosis of CRC negatively affects patient quality of life (QoL). Research demonstrates that diagnosing and treating CRC cause long-term psychological, physical, social, and functional consequences, which may impair QoL [3,4].

Risk factors in CRC patients associated with poorer QoL and higher psychological stress include advanced disease stage, presence of a stoma, asthenia, lack of a partner, lower income, and smoking [5,6].

Malignant tumors, including CRC, particularly in advanced stages, are often associated with a high physical burden and severe metabolic disturbances leading to cachexia, which substantially impacts QoL. From a clinical perspective, cancer cachexia is characterized by chronic, unintended, and progressive weight loss, very often accompanied by anorexia, early satiety, and asthenia [7].

There is no universally accepted definition of cachexia. Fearon K.C. et al. distinguished three stages: pre-cachexia, cachexia, and refractory cachexia [7]. The Working Scrinio Group defines pre-cachexia as unintended weight loss < 10%, and cachexia as weight loss ≥ 10% accompanied by anorexia, asthenia, or early satiety [8]. Cachexia develops gradually, and when diagnosed late, it leads to severe and often irreversible wasting. Therefore, its early detection is crucial. During the pre-cachexia stage, nutritional interventions, including oral nutritional supplements (ONS), may significantly improve nutritional status and QoL [9].

CRC requires a complex approach, as the diagnosis affects multiple aspects of a patient’s life including personal, professional, social, and family-related domains [10]. As a result, health-related quality of life in this group of patients may be markedly impaired [11,12]. HRQoL reflects the impact of disease and treatment on physical, social, family, emotional, and functional functioning, as well as on nutrition-related concerns and cancer-specific symptoms [13,14].

Various tools are used to evaluate QoL in cancer patients, with the most commonly used being the EORTC QLQ-C30 (European Organisation for Research and Treatment of Cancer) and the Functional Assessment of Cancer Therapy (FACT) in versions dedicated to specific types of malignancy [9].

The Functional Assessment of Anorexia/Cachexia Therapy (FAACT) enables a comprehensive evaluation of symptoms associated with cancer cachexia, such as anorexia, fatigue, reduced physical activity, and emotional disturbances [15].

Despite the recognized importance of HRQoL in the treatment of CRC, current interventions remain focused mainly on symptom management, primarily through pharmacotherapy, chemotherapy, or radiotherapy. In a comprehensive approach to CRC therapy, the goal should not only be clinical remission but also achieving high QoL at each stage of treatment. A multidimensional perspective that incorporates the patient’s subjective experience across different areas of life is therefore essential. This approach is regarded not only as an important indicator of care quality but also as a prognostic factor for mortality, as higher HRQoL has been linked to lower risk of death [16].

## 2. The Aims of the Study

Primary aim. To evaluate health-related quality of life (HRQoL) and performance status in patients with colorectal cancer (CRC) undergoing chemotherapy who were eligible for oral nutritional supplements (ONS).

Secondary aim. To investigate the relationships between sex, nutritional status, performance status, and appetite, and their associations with HRQoL.

## 3. Materials and Methods

The present study included an in-depth sub-analysis of quality of life in a cohort of patients recruited as part of the study described in the publication: Chemotherapy-Related Toxicity, Nutritional Status and Quality of Life in Precachectic Oncologic Patients with, or without, High Protein Nutritional Support. A Prospective, Randomized Study, Nutrients 2017, 9 (10), 1108 [17]. Only patients who had fully completed all quality of life assessment forms were included in the analysis. In this subgroup, relationships between quality-of-life, functional status, and nutritional status were evaluated.

A total of 72 patients (36 men, 36 women) with colorectal cancer (CRC) were included. The study was approved by the Bioethics Committee of the Medical University of Gdańsk (approval number NKBBN/412/2014). This study was registered at ClinicalTrials.gov (Identifier: NCT02848807; registration date: 27 July 2016). Written informed consent was obtained from all participants. The study was conducted at the Department of Oncology and Radiotherapy, Medical University of Gdańsk.

**Figure 1:**
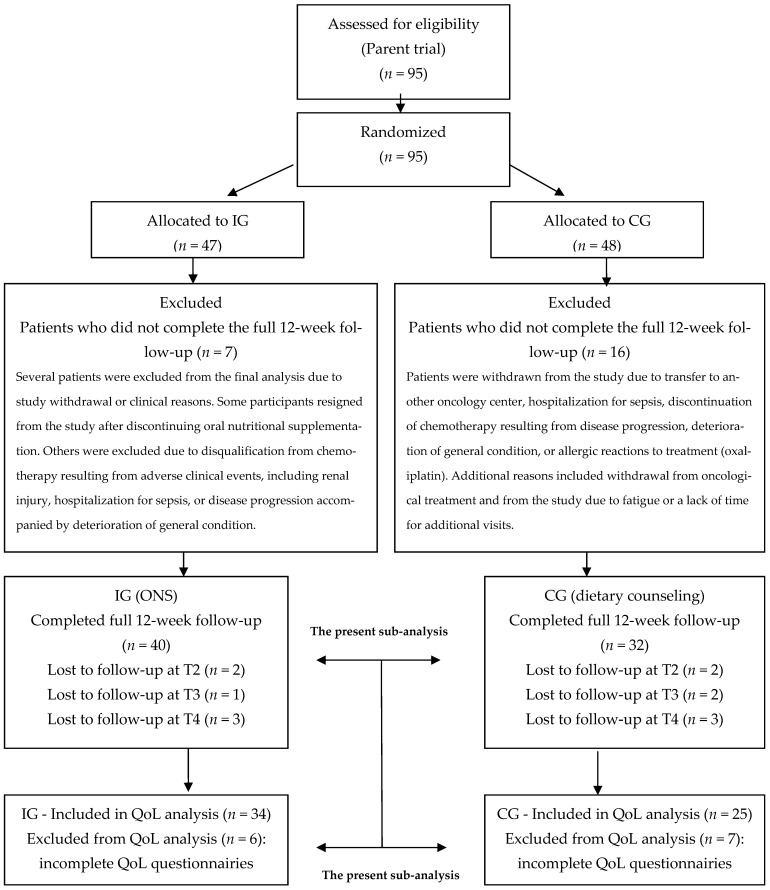
CONSORT-style flow diagram illustrating patient recruitment, randomization, follow-up, and inclusion in the quality-of-life analysis.

Using computer-assisted randomization, 40 patients were assigned to the intervention group (IG) and 32 to the control group (CG) (study design is presented in Figure 1).

A CONSORT-style flow diagram was used to illustrate patient recruitment, randomization, follow-up, and inclusion in the quality-of-life analysis. Only patients with complete QoL data at all assessment timepoints were included in the final analyses.

### 3.1. Relationship to Parent Study and Selection of QoL Subsample

This manuscript presents a secondary (sub-)analysis of a randomized interventional study that enrolled 95 patients with colorectal cancer and randomized them to either the intervention group (oral nutritional supplementation, ONS) or the control group (dietary counseling). The processes of patient recruitment, randomization, and follow-up are presented in a CONSORT-style flow diagram (Figure 1).

Of the 95 patients included in the parent study, a proportion did not complete the whole 12-week follow-up period due to clinical or organizational reasons, including withdrawal of consent, disease progression, hospitalization, treatment-related complications, transfer to another oncology center, or voluntary discontinuation of study participation. Detailed reasons for exclusion at each follow-up stage are provided in the CONSORT diagram.

Ultimately, 82 patients completed the whole 12-week observation period. All of these patients completed quality-of-life questionnaires at baseline (T1). However, not all participants provided complete quality-of-life data at all subsequent assessment timepoints (T2, T3, and T4). Therefore, only patients who completed QoL questionnaires at all assessment points were included in the final quality-of-life analysis.

As a result, the quality-of-life statistical analysis included 34 patients from the intervention group and 25 from the control group. The remaining patients were excluded from the QoL analysis due to incomplete questionnaire data. This complete-case analysis approach allowed for consistent longitudinal comparisons; however, it is associated with a potential risk of selection bias, which is addressed in the Discussion section.

### 3.2. Characteristics of the Study Group

The mean age of patients in the intervention group (IG) was 72.62 ± 10.56 years, and in the control group (CG), it was 71.28 ± 11.68 years. Colostomy was reported in 34 patients in the IG and 21 patients in the CG.

Histopathological confirmation of colorectal cancer was obtained in all participants. According to the TNM classification, 32 patients (44.4%) were diagnosed with stage IV disease, 36 patients (50%) with stage III, and 4 patients (5.6%) with stage II. Only 7 patients (9.7%) had no metastases. Lymph node involvement was diagnosed in 35 patients (48.6%). Distant metastases (lungs, ovaries, liver, bladder, kidneys, adrenal glands) were found in 30 patients (41.7%). Sixty-three patients (87.5%) were in clinical stage G2, 7 patients (9.7%) in G3, and 2 patients (2.8%) in G4.

Tumor localization was as follows: cecum: 4 patients (5.6%); colon (ascending, transverse, descending, sigmoid): 38 patients (52.8%); rectum: 30 patients (41.6%). All patients were eligible for chemotherapy. A total of 33.0% of patients qualified for the FOLFOX regimen, while the remainder were eligible for FOLFIRI, LF, CLF, or XELOX. Baseline demographic and clinical characteristics were generally comparable between the CG and IG. No significant differences were observed in age (*p* = 0.683), sex distribution (*p* = 0.155), tumor grade (*p* = 0.305), metastasis frequency (*p* = 0.096), or chemotherapy regimen allocation (*p* = 0.765).

The only statistically significant difference between groups concerned the presence of colostomy, which was more frequent in the intervention group (52.5% vs. 18.8%, *p* = 0.003) (Table 1).

**Nutritional Intervention**

Patients in the IG received a hypercaloric, high-protein ONS (2 bottles of 125 mL/day), for the entire 12-week observation period. Each serving provided 300 kcal and 18 g protein. The supplement was nutritionally complete, containing all macronutrients, vitamins, and minerals, and was lactose- and gluten-free. It was available in four flavors, allowing patients to choose according to their preference.

Data were collected over 12 weeks of observation, with follow-up visits every 4 weeks. At each visit, quality of life was assessed using the Functional Assessment of Anorexia/Cachexia Therapy questionnaire and the Karnofsky Performance Status Scale. Nutritional status, compliance with recommendations, and tolerance of oral nutritional supplements (ONS) were also monitored.

In IG, to assess adherence, patients were provided with a defined number of ONS bottles and were asked to return unused containers at the next visit. In addition, patients kept a daily record of the number of consumed portions on a specially prepared form.

### 3.3. Inclusion Criteria

1. Histologically confirmed diagnosis of CRC, clinical stage II–IV according to TNM UICC 2010 [18].
2. Eligibility for first-line chemotherapy according to regimens including: 5-fluorouracil, leucovorin, oxaliplatin (FOLFOX-4) or 5-fluorouracil, leucovorin, irinotecan (FOLFIRI), and other regimens; full chemotherapy dose (no dose reduction).
3. Performance status ≥ 80% on the Karnofsky scale.
4. Asymptomatic pre-cachexia associated with cancer, diagnosed according to the SCRINIO Working Group.
5. No contraindications to oral nutrition and practical feasibility of oral feeding.
6. No severe, uncontrolled comorbidities such as diabetes, liver failure, or renal failure (stage 2 according to KDOQI).
7. Signed informed consent for participation.

### 3.4. Exclusion Criteria

1. Diagnosis of malignant neoplasm in clinical stage I according to TNM UICC 2010 [18].
2. Disqualification from oncological treatment.
3. Overt cancer cachexia or anorexia–cachexia syndrome.
4. Poor performance status—Karnofsky scale < 80% or WHO/ECOG 2–4.
5. Contraindications to oral feeding or high-protein nutrition (e.g., liver or renal failure).
6. Regular nutritional support at the time of study enrollment.
7. Patient non-compliance at the time of study enrollment.

### 3.5. Quality of Life

#### 3.5.1. FAACT

Quality of life was assessed using the FAACT questionnaire, Version 4.0 [19], which evaluates both general aspects of QoL and symptoms associated with anorexia/cachexia syndrome. It includes the 27-item Functional Assessment of Cancer Therapy–General (FACT-G) and a 12-item anorexia/cachexia subscale (ACS). In total, 39 questions are grouped into five subscales: Physical Well-Being (PWB) (7 items, score range: 0–28), Social/Family Well-Being (SWB) (7 items, 0–28), Emotional Well-Being (EWB) (6 items, 0–24), Functional Well-Being (FWB) (7 items, 0–28), and Additional Concerns—Anorexia/Cachexia Subscale (ACS) (12 items, 0–48).

The questionnaire requires approximately 10–15 min to complete. Responses refer to the last 7 days and are scored on a Likert-type scale: 0 = Not at all; 1 = A little bit; 2 = Somewhat; 3 = Quite a bit; 4 = Very much. Some items are reverse-scored, as specified in the FAACT scoring manual.

- Subscale scores are calculated by summing the item scores, multiplying by the number of items in the subscale, and dividing by the number of items answered.
- The Trial Outcome Index (TOI) is obtained by summing PWB, FWB, and ACS scores.
- The FACT-G total score is obtained by summing PWB, SWB, EWB, and FWB scores.
- The FAACT total score is obtained by summing PWB, SWB, EWB, FWB, and ACS scores.

The maximum possible FAACT score is 156; lower scores indicate poorer overall QoL. Results were analyzed according to the FAACT scoring guidelines [20].

#### 3.5.2. Karnofsky Scale

The Karnofsky Performance Status (KPS) scale is a widely used tool for assessing functional status in oncology patients. It is a percentage scale (0–100%), where higher values indicate better physical function and higher QoL. The scale reflects only one dimension of QoL—physical functioning. A score of 100 corresponds to normal health without disease symptoms, 90 to normal activity with minor symptoms, 80 to near-normal activity requiring some effort, and progressively lower scores correspond to increasing disability, requiring partial or complete care, down to 0, which indicates death.

#### 3.5.3. Nutritional Status

Nutritional status and appetite were also assessed. At each visit, body weight was measured using a body composition analyzer (Tanita BC 420), and body mass index (BMI) was calculated as weight/height^2^ (kg/m^2^).

Nutritional status was also evaluated using Nutritional Risk Screening 2002 (NRS-2002) and the seven-point Subjective Global Assessment (SGA).

- NRS-2002 includes three categories: impaired nutritional status (assessed by BMI, unintentional weight loss, and food intake relative to needs), disease severity, and age. A score ≥ 3 indicates nutritional risk and the need for nutritional intervention.
- SGA includes five categories: weight history, dietary intake, gastrointestinal symptoms, functional capacity, disease/comorbidities relative to nutritional needs, and physical examination. Each is rated on a 1–7 scale. Patients are classified as: well nourished (6–7), moderately malnourished (3–5), or severely malnourished (1–2).

Appetite was assessed using a visual analogue scale (VAS) referring to the previous 7 days. The scale is a 100 mm line, where 0 = no appetite and 100 = very good appetite. The score corresponds to the distance (mm) the patient marked. Scores < 70 mm indicated moderate appetite loss, and scores < 50 mm indicated severe appetite loss.

### 3.6. Statistical Analysis

Statistical analysis was performed using Statistica version 13.3 (StatSoft, Tulsa, OK, USA) and Microsoft Excel 365 (Microsoft Corporation, Redmond, WA, USA). Figures were prepared using GraphPad Prism version 8.4.3 (GraphPad Software, San Diego, CA, USA). A *p*-value < 0.05 was considered statistically significant.

Descriptive statistics included means, standard deviations (SD), medians, and minimum–maximum values for quantitative variables, and frequencies (*n*) with percentages (%) for categorical variables.

Data normality was assessed using the Shapiro–Wilk test [21]. Changes over time (T1–T4) within groups were evaluated with repeated-measures ANOVA for normally distributed variables and the Friedman test [22] for non-normal distributions.

Between-group comparisons (intervention vs. control) used independent samples t-tests for normally distributed data and Mann–Whitney U tests [23] otherwise.

Baseline characteristics were compared using the Mann–Whitney U test [23] for age, Pearson’s χ^2^ test [24] for sex and chemotherapy regimen, Fisher–Freeman–Halton exact test [25] for tumor grade, and Fisher’s exact test [26] for metastases and colostomy (applied when expected cell counts were <5).

Minimally important differences (MID) for FAACT scores were calculated via a distribution-based approach, defined as 0.5 × SD of baseline scores [27,28]. Patients were classified as improved (change ≥ MID) or deteriorated (change ≤ −MID).

Proportions achieving MID thresholds were compared using Pearson’s χ^2^ test [24] or Fisher’s exact test [26] (for expected counts < 5), with odds ratios (ORs) and 95% confidence intervals (CIs) reported.

Associations between QoL (FAACT scores), nutritional status (SGA, NRS-2002), functional performance (Karnofsky scale), and appetite (VAS) were examined using Pearson [29] or Spearman [30] correlation coefficients based on data distribution.

Confidence intervals (95% CI) for binomial proportions are estimated using the Wilson score interval [31]. For means in *t*-tests and ANOVA, the t-distribution is applied [32], for large samples, the z-test uses the normal distribution with z = 1.96 [33]. Non-parametric methods include the Hodges-Lehmann estimator for median differences in Wilcoxon tests [34] and the Woolf logit method for odds ratios in contingency tables [35]. For Spearman correlations, 95% CIs were computed using the Fieller et al. [36] correction [37].

Effect sizes quantified the magnitude of effects: Cohen’s d [38] for *t*-tests (small: ≥ 0.20; medium: ≥0.50; large: ≥0.80); eta squared (η^2^) (Cohen, 1988) for non-paired ANOVA (small: ≥0.01; medium: ≥0.06; large: ≥0.14); Kendall’s coefficient of concordance (*W*) [39] for Friedman tests (small: ≥0.10; medium: ≥0.30; large: ≥0.50); rank-biserial correlation (*r*) [40] for Mann–Whitney U tests (small: ≥0.10; medium: ≥0.30; large: ≥0.50); phi (*φ*) for 2 × 2 χ^2^ tests or Cramér’s *V* [41] for larger tables (small: ≥0.10; medium: ≥0.30; large: ≥0.50); Spearman’s rho (ρ) [30] for correlations (small: |ρ| ≥ 0.10; medium: ≥0.30; large: ≥0.50); and ORs for binary outcomes (small: ≥1.68; medium: ≥3.47; large: ≥6.71) [42].

Due to the exploratory nature of the study, uncorrected *p*-values were reported for multiple comparisons [43].

Analyses employed a complete-case approach, including only fully completed QoL questionnaires; no imputation was performed. Reverse-scored FAACT items followed instrument guidelines.

## 4. Results

### 4.1. FAACT Results—Total Group

**Figure 2:**
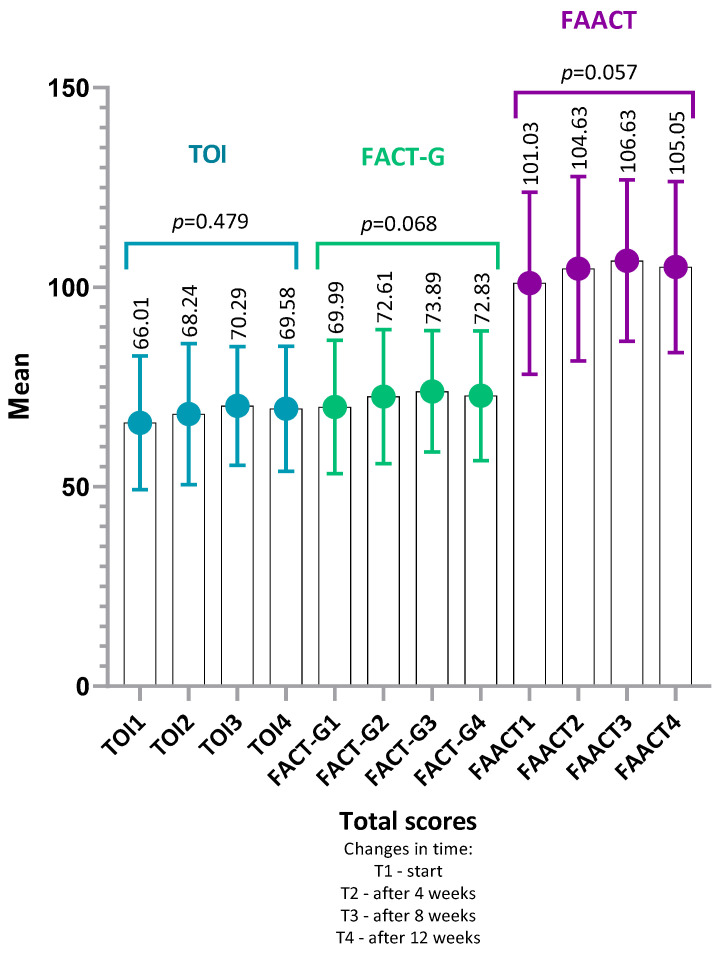
Changes in TOI, FACT-G, and FAACT total scores over time. Mean values (±SD) are presented for TOI, FACT-G and FAACT at baseline (T1), 4 weeks (T2), 8 weeks (T3), and 12 weeks (T4). *p*-values refer to within-group changes over time and were calculated using Friedman ANOVA with Kendall’s coefficient of concordance (W). No statistically significant differences were observed, although FACT-G (*p* = 0.07) and FAACT (*p* = 0.06) showed a trend toward improvement. Abbreviations: TOI, Trial Outcome Index; FACT-G, Functional Assessment of Cancer Therapy–General; FAACT, Functional Assessment of Anorexia/Cachexia Therapy.

The mean total FAACT score at baseline was 101.0 ± 22.8 (95% CI: 94.6–107.4, median 100, range: 56.0–152.0). After 12 weeks of observation (T4), this score increased to 105.1 ± 21.4 (95% CI: 99.1–111.1, median 103.0, range: 57.0–155.0). The results indicated reduced quality of life (QoL) across all categories and time points (four assessments every four weeks). No statistically significant differences were observed in mean FAACT scores during the entire observation period (*p* = 0.06, W = 0.05) (Figure 2). Although this change did not reach statistical significance, it may indicate a trend toward improved subjective QoL during the intervention.

Analysis of partial indices—FACT-G (sum of PWB, SWB, EWB, FWB) and the Trial Outcome Index (TOI) (sum of PWB, FWB, ACS)—also showed no significant changes over time (*p* = 0.07, W = 0.05 and *p* = 0.48, W = 0.02, respectively) (Figure 2).

**Figure 3:**
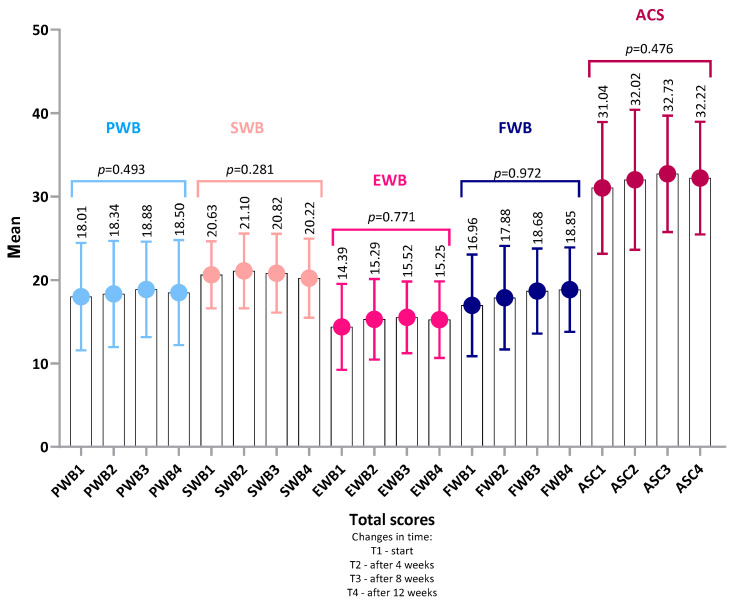
Changes in FACT-G subscales and ACS scores over time. Mean values (±SD) are presented for PWB, SWB, EWB, FWB, and ACS at baseline (T1), 4 weeks (T2), 8 weeks (T3), and 12 weeks (T4). *p*-values refer to within-group changes over time and were calculated using Friedman ANOVA with Kendall’s coefficient of concordance (W). No statistically significant differences were observed. Abbreviations: PWB, Physical Well-Being; SWB, Social/Family Well-Being; EWB, Emotional Well-Being; FWB, Functional Well-Being; ACS, Additional Concerns—Anorexia/Cachexia Subscale.

Similarly, no significant differences were observed in the total values of each of the five FAACT subscales (Figure 3): PWB, SWB, EWB, FWB, and ACS.

Taken together, the mean FAACT scores in the total sample—both in the overall assessment and across each subscale—remained at levels indicating reduced QoL in patients undergoing chemotherapy. These results suggest a substantial physical and emotional burden already present at the time of study enrollment. The high standard deviations in individual assessments may further indicate heterogeneity of responses in the study group and the existence of subgroups of patients with different adaptive profiles (Figure 2 and Figure 3).

The mean subscale score was highest in the Social/Family Well-Being (SWB) category (T1: 20.63 ± 4.03, 95% CI: 19.50–21.76; T4: 20.22 ± 4.74, 95% CI: 18.89–21.55) and lowest in the Emotional Well-Being (EWB) category (T1: 15.39 ± 5.15, 95% CI: 13.94–16.84; T4: 15.25 ± 4.60, 95% CI: 13.96–16.54), both at baseline and at the end of observation. This indicates that, alongside the overall trend toward improvement in QoL, participants rated social and family relationships as the highest, whereas the emotional domain was the weakest aspect of QoL in this cohort.

#### 4.1.1. Analysis of Item-Level Results Within Each Category FAACT

No statistically significant differences were observed for individual items in the PWB category across the entire observation period (GP1–GP7: *p* > 0.05: GP1: *p* = 0.20, W = 0.03; GP2: *p* = 0.06, W = 0.05; GP3: *p* = 0.66, W = 0.01; GP4: *p* = 0.97, W = 0.00; GP5: *p* = 0.91, W = 0.00; GP6: *p* = 0.68, W = 0.01; GP7: *p* = 0.38, W = 0.02).

Similarly, no significant differences were noted in the FWB category (GF1–GF7: *p* > 0.05: GF1: *p* = 0.18, W = 0.03; GF2: *p* = 0.90, W = 0.00; GF3: *p* = 0.88, W = 0.00; GF4: *p* = 0.60, W = 0.01; GF5: *p* = 0.43, W = 0.02; GF6: *p* = 0.82, W = 0.01; GF7: *p* = 0.22, W = 0.03). These findings suggest that despite ongoing treatment and the passage of time, there were no major changes in the subjective perception of symptoms such as pain, nausea, or general well-being (GP2, GP4, GP6), nor in functional aspects such as ability to work, sleep quality, or enjoyment of life (GF1–GF7). This may indicate stability or the chronic nature of these limitations within the study population.

In the SWB category, which includes aspects of social support, interpersonal relationships, and family communication, no significant changes were observed over time (GS1–GS7: *p* > 0.05: GS1: *p* = 0.61, W = 0.01; GS2: *p* = 0.27, W = 0.02; GS3: *p* = 0.38, W = 0.02; GS4: *p* = 0.09, W = 0.04; GS5: *p* = 0.06, W = 0.04; GS6: *p* = 0.47, W = 0.01; GS7: *p* = 0.73, W = 0.01). Stability of scores (*p* > 0.05) suggests that the perception of social support and family relationships remained relatively unchanged. However, GS4 and GS5 (family acceptance of illness and communication about the disease) showed *p*-values close to statistical significance (*p* = 0.09 and *p* = 0.06, respectively), suggesting a possible trend toward improvement in these domains. This may reflect a gradual, albeit modest, increase in perceived support and understanding within family relationships.

In the EWB category, no significant changes were found in 5 out of 6 items (GE1, GE3–GE6; *p* > 0.05: GE1: *p* = 0.15, W = 0.03; GE3: *p* = 0.23, W = 0.02; GE4: *p* = 0.58, W = 0.01; GE5: *p* = 0.39, W = 0.02; GE6: *p* = 0.58, W = 0.01). These items addressed sadness (GE1), loss of hope (GE3), anxiety (GE4), fear of death (GE5), and concerns about worsening health (GE6), which may indicate sustained emotional burden associated with the disease.

An exception was item GE2 (“I am satisfied with how I am coping with my illness”), which showed a significant improvement (*p* = 0.003, W = 0.09). Post hoc Durbin-Conover tests confirmed that satisfaction with coping was significantly higher at 12 weeks (T4) compared with baseline (T1, *p* = 0.006, r = 0.36; T2, *p* = 0.020, r = 0.33). This suggests that despite persistent emotional distress, patients developed greater confidence and a sense of control over their illness, potentially due to adaptation, therapeutic support, or a perception of effective treatment (chemotherapy).

In the ACS (Additional Concerns Subscale), which addresses cachexia-related symptoms and nutritional issues, no significant changes were noted in 11 out of 12 items (*p* > 0.05: C6: *p* = 0.86, W = 0.00; ACT1: *p* = 0.23, W = 0.02; ACT2: *p* = 0.17, W = 0.03; ACT3: *p* = 0.12, W = 0.03; ACT4: *p* = 0.66, W = 0.01; ACT6: *p* = 0.90, W = 0.00; ACT7: *p* = 0.31, W = 0.02; ACT9: *p* = 0.16, W = 0.03; O2: *p* = 0.65, W = 0.01; ACT10: *p* = 0.62, W = 0.01; ACT11: *p* = 0.63, W = 0.01). This stability suggests persistent challenges related to appetite, eating difficulties, concerns about body weight, vomiting, and early satiety throughout the study period.

The only exception was item ACT13 (“My overall health is improving”), for which significantly higher scores were observed at 8 weeks (T3) compared with baseline (T1 and T2; *p* = 0.049, r = 0.30 and *p* = 0.02, r = 0.34, respectively). This increase in self-reported health may reflect a subjective sense of improvement associated with therapeutic measures or supportive care during observation.

#### 4.1.2. Minimally Important Differences (MID)—FAACT

MID is defined as “the smallest difference in score in the domain of interest that patients perceive as important, either beneficial or harmful, and that would lead the clinician to consider a change in the patient’s management” [44].

The analysis of minimally important differences (MID), based on a statistical approach (0.5 × standard deviation), for FAACT and its subscales indicated that a substantial proportion of patients experienced meaningful changes in QoL during the observation period. MID represents the minimal change in score that patients perceive as necessary and that may influence clinical decisions.

For the total FAACT score, the MID was 11.41 points; 13 patients experienced improvement above this threshold, while 8 patients reported deterioration by at least this margin. For FACT-G (MID = 8.35), improvement was observed in 14 patients, whereas 8 patients experienced deterioration (Table 2).

The highest proportion of patients showing improvement was observed in the ACS (*n* = 16), related to nutritional problems and cachexia, and in the EWB subscale (*n* = 14), reflecting emotional coping with the disease. Conversely, the greatest proportion of patients reporting deterioration in QoL was noted in SWB (11 patients) and PWB (10 patients), which may reflect difficulties in social relationships and persistent physical symptoms. These findings suggest that, despite an overall trend toward improvement, some patients experienced deterioration in selected QoL domains, highlighting the importance of individualized therapeutic support.

Comparison between the intervention group (IG) and control group (CG) (Table 3) showed that:

- FAACT (global score): improvement was significantly more frequent in IG (32%) than in CG (8%) (*p* = 0.03, OR = 5.50, 95% CI: 1.10–27.62, φ = 0.25).
- PWB (Physical Well-Being): improvement was significantly more frequent in IG (32%) than in CG (8%) (*p* = 0.03, OR = 5.50, 95% CI: 1.10–27.62, φ = 0.25).
- EWB (Emotional Well-Being): improvement was markedly more frequent in IG (38%) than in CG (4%) (*p* = 0.002, OR = 14.86, 95% CI: 1.79–123.36, φ = 0.36).

No statistically significant differences were observed in other scales (FACT-G, SWB, FWB, ACS). However, it is worth noting that:

- In FWB, the proportion of improvement in IG (29%) was higher than in CG (8%), with results approaching significance (*p* = 0.05, OR = 4.79, 95% CI: 0.95–24.27, φ = 0.22).
- In ACS, deterioration was less frequent in IG (3%) compared with CG (20%), also reaching borderline significance (*p* = 0.07, OR = 0.12, 95% CI: 0.01–1.11, φ = 0.22).

In summary, the intervention produced the most significant benefits in global quality of life (FAACT; OR = 5.5, φ = 0.29), physical well-being (PWB; OR = 5.5, φ = 0.29), and emotional well-being (EWB; OR = 14.86, φ = 0.40). In other domains, favorable trends were observed, although they did not reach statistical significance.

### 4.2. FAACT Results—IG vs. CG

Analysis of mean total FAACT scores in the intervention group (IG) and control group (CG) showed that quality of life was reduced at baseline and remained at a similar level throughout the 12-week study period, without statistically significant changes over time.

In IG, the mean total FAACT score at baseline was 98.43 ± 20.98 (95% CI: 90.86–105.99; median: 97.5, range: 57.0–148.0), while at the end of observation (12 weeks), it was 105.94 ± 18.8 (95% CI: 99.15–112.73; median: 106.5, range: 66.0–141.0). Despite this upward trend, the difference did not reach statistical significance (*p* = 0.09); however, the associated effect size corresponded to a medium magnitude (r = 0.30). This suggests that the observed improvement in the intervention group may be clinically relevant and that the lack of statistical significance could be attributable to insufficient statistical power rather than the absence of an intervention effect.

In CG, the mean total FAACT score at baseline was 104.28 ± 24.88 (95% CI: 93.99–114.57; median: 101.0, range: 56.0–152.0), and after 12 weeks it was 103.84 ± 24.92 (95% CI: 93.53–114.15; median: 100.0, range: 57.0–155.0), with no significant change over time (*p* = 0.28, r = 0.22).

Similarly, no statistically significant changes over time were observed in:

- FACT-G score (IG: *p* = 0.22, r = 0.22; CG: *p* = 0.14, r = 0.30),
- TOI (Trial Outcome Index) (IG: *p* = 0.17, r = 0.24; CG: *p* = 0.29, r = 0.22),
- or in any of the FAACT subscales: PWB, SWB, EWB, FWB, ACS (all *p* > 0.05 for both groups).

Thus, the results of each FAACT category did not change significantly over time in either group when analyzed separately.

**Figure 4:**
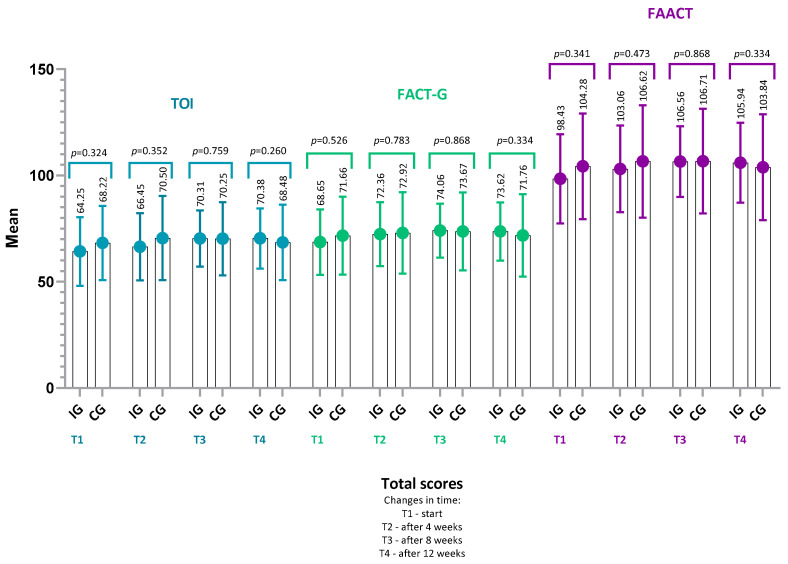
Comparison of TOI, FACT-G, and FAACT scores between the intervention group (IG) and the control group (CG) over 12 weeks. Mean values (±SD) are presented for TOI, FACT-G, and FAACT at baseline (T1), 4 weeks (T2), 8 weeks (T3), and 12 weeks (T4). *p*-values refer to between-group comparisons (IG vs. CG) and were calculated using the Mann–Whitney U test. No statistically significant differences were observed. Abbreviations: IG, intervention group; CG, control group; TOI, Trial Outcome Index; FACT-G, Functional Assessment of Cancer Therapy–General; FAACT, Functional Assessment of Anorexia/Cachexia Therapy.

**Figure 5:**
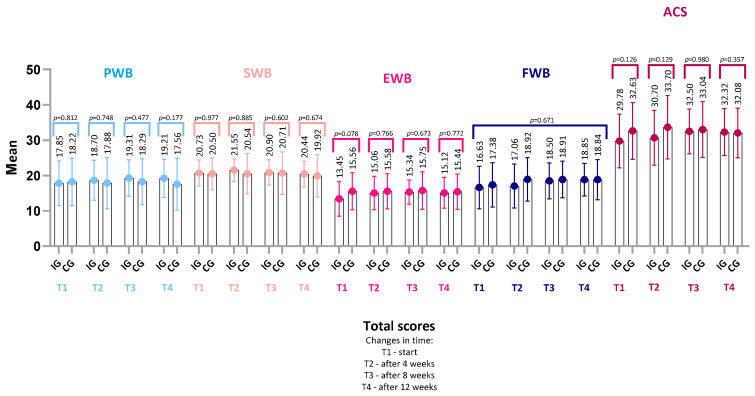
Comparison of FAACT subscale scores between the intervention group (IG) and the control group (CG) over 12 weeks. Mean values (±SD) are presented for PWB, SWB, EWB, FWB, and ACS at baseline (T1), 4 weeks (T2), 8 weeks (T3), and 12 weeks (T4). Between-group comparisons (IG vs. CG) were performed using the Mann–Whitney U test for PWB, SWB, EWB, and ACS, and repeated-measures ANOVA for FWB. No statistically significant differences were observed. Abbreviations: PWB, Physical Well-Being; SWB, Social/Family Well-Being; EWB, Emotional Well-Being; FWB, Functional Well-Being; ACS, Additional Concerns—Anorexia/Cachexia Subscale.

Moreover, between-group comparisons (IG vs. CG) showed no statistically significant differences across the entire observation period for any of the assessed parameters: total FAACT score (*p* > 0.05), FACT-G score (*p* > 0.05), TOI (*p* > 0.05), or individual subscales (PWB, SWB, EWB, FWB, ACS; all *p* > 0.05): Figure 4 and Figure 5.

Although a slight increase in the mean FAACT score was observed in IG, this change did not reach statistical significance, indicating only a trend toward improvement in quality of life during the 12-week study period.

### 4.3. FAACT Results—Women vs. Men

**Figure 6:**
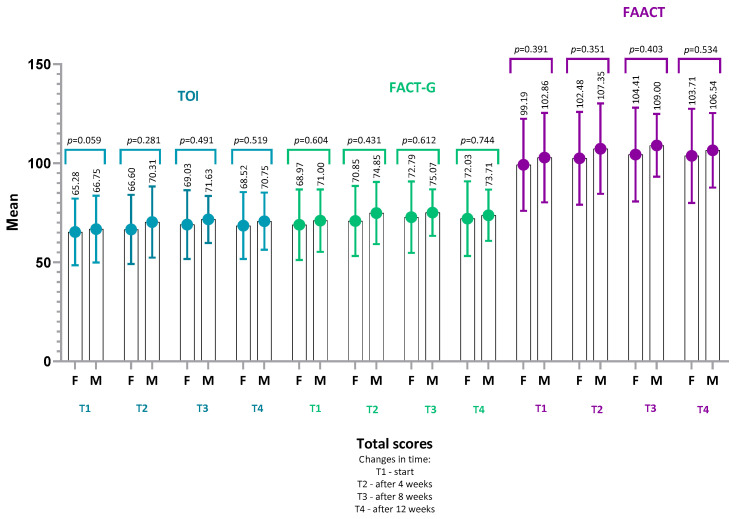
Comparison of TOI, FACT-G, and FAACT scores between females (F) and males (M) over 12 weeks. Mean values (±SD) are presented for TOI, FACT-G, and FAACT at baseline (T1), 4 weeks (T2), 8 weeks (T3), and 12 weeks (T4). Between-group comparisons (female vs. male) were performed using the Mann–Whitney U test. No statistically significant differences were observed. Abbreviations: TOI, Trial Outcome Index; FACT-G, Functional Assessment of Cancer Therapy–General; FAACT, Functional Assessment of Anorexia/Cachexia Therapy.

**Figure 7:**
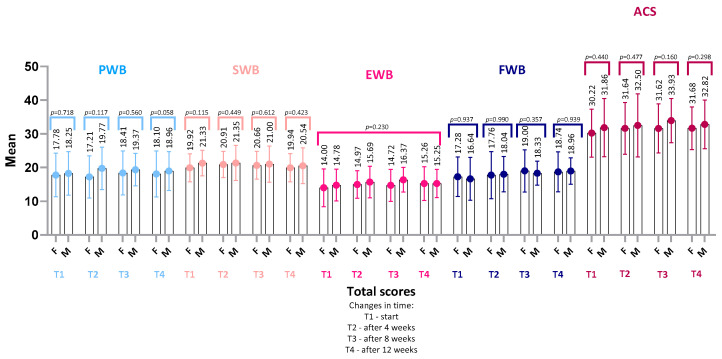
Comparison of FAACT subscale scores between females (F) and males (M) over 12 weeks. Mean values (±SD) are presented for PWB, SWB, EWB, FWB, and ACS at baseline (T1), 4 weeks (T2), 8 weeks (T3), and 12 weeks (T4). Between-group comparisons (female vs. male) were performed using the Mann–Whitney U test, except for EWB, which was analyzed with repeated-measures ANOVA. No statistically significant differences were observed. Abbreviations: PWB, Physical Well-Being; SWB, Social/Family Well-Being; EWB, Emotional Well-Being; FWB, Functional Well-Being; ACS, Additional Concerns—Anorexia/Cachexia Subscale.

Comparative analysis of quality-of-life (FAACT) scores between women and men showed no statistically significant differences at any of the assessed time points (Figure 6). In both groups, mean FAACT scores and their subscales (PWB, SWB, EWB, FWB, ACS) remained stable over time, without significant within-group or between-group differences (*p* > 0.05) (Figure 7).

At baseline (T1), QoL scores were slightly lower in women (mean FAACT: 99.2 ± 23.2, 95% CI: 89.7–108.7) compared with men (102.9 ± 22.6, 95% CI: 93.6–112.2), but these differences were not statistically significant (*p* = 0.39, r = 0.11). A similar pattern was observed at the end of the study (T4), where women scored 103.7 ± 23.8 (95% CI: 93.9–113.5) and men 106.5 ± 18.8 (95% CI: 99.0–114.0; *p* = 0.53, r = 0.08).

In the Physical Well-Being (PWB) subscale, some differences in mean values between women and men were noted, but these also did not reach statistical significance (e.g., PWB at T4: women 18.1 ± 6.8, 95% CI: 15.3–20.9 vs. men 19.0 ± 5.8, 95% CI: 16.6–21.4; *p* = 0.06, r = 0.25). These trends suggest a similar profile of QoL and perception of disease-related symptoms in both sexes, which may be explained by comparable levels of functional performance (KPS).

The lack of significant differences indicates that sex was not a differentiating factor for general QoL in this cohort of CRC patients with pre-cachexia.

### 4.4. Performance Status—Karnofsky Scale

At baseline, most patients demonstrated high functional performance according to the Karnofsky Performance Status (KPS) scale: 52.8% scored 90, and 34.7% achieved the maximum score of 100, indicating minimal or no disease-related symptoms and the ability to perform routine daily activities. Over the 12-week observation period, the distribution of scores remained stable (Table 4). The percentage of patients scoring 90 or 100 did not change significantly (T1: 87.5%, 95% CI: 77.6–93.2; T4: 86.5%, 95% CI: 75.7–92.8), while the proportion of patients with moderate functional limitation (score 80) increased slightly from 11.1% (95% CI: 5.7–20.4) to 13.5% (95% CI: 7.0–24.5). At the same time, the number of patients with a score of 70 declined to zero. The mean score in the entire group at T1 was 92.08 ± 6.91 (95% CI: 90.46–93.70; median: 90.0; range: 70–100). In subsequent assessments, mean performance levels remained similar: T2—92.09 ± 6.86 (95% CI: 90.42–93.76); T3—91.67 ± 7.85 (95% CI: 89.64–93.70); T4—92.04 ± 6.64 (95% CI: 90.31–93.77). These differences were not statistically significant (*p* = 0.37, W = 0.02), indicating stability of physical functioning throughout the observation period (Table 5). Subgroup analyses also revealed no statistically significant changes over time. In CG, mean values across T1–T4 ranged from 92.50 ± 6.76 (95% CI: 89.71–95.29) to 94.14 ± 5.68 (95% CI: 91.80–96.48), with no significant changes over time (*p* = 0.12, W = 0.04). In IG, mean values ranged from 90.29 ± 8.57 (95% CI: 87.30–93.28) to 91.71 ± 6.64 (95% CI: 89.39–94.03), with no significant changes across successive assessments (*p* = 0.52, W = 0.01). Importantly, comparisons between CG and IG showed no statistically significant differences at any time point: T1 (*p* = 0.08, r = 0.23), T2 (*p* = 0.13, r = 0.20), T3 (*p* = 0.13, r = 0.20), T4 (*p* = 0.65, r = 0.06)—Table 5.

Performance status assessed with the Karnofsky scale remained stable throughout the 12-week observation period in both IG and CG. The lack of significant differences between groups suggests that ONS did not have a significant impact on overall functional performance in the study population during the evaluated period. The stability of values may reflect both the intervention’s limited effect and the ability to maintain good functional status despite disease and treatment.

### 4.5. Nutritional Status

Assessment of nutritional status using the SGA and NRS-2002 scales revealed heterogeneity in the study population, ranging from well-nourished patients to those showing features of malnutrition. Although significant baseline differences in nutritional status were observed between CG and IG (with worse nutritional status in CG), no statistically significant differences were found between groups at the end of observation (T4) when assessed with SGA (*p* = 0.33, r = 0.12), NRS-2002 (*p* = 0.82, r = 0.03), or BMI (*p* = 0.16, r = 0.18).

In contrast to CG, patients in IG demonstrated an improvement in nutritional status during the 12-week observation period (SGA: 4.63 ± 0.95 [95% CI: 4.30–4.96] at T1 vs. 5.43 ± 0.56 [95% CI: 5.23–5.63] at T4, *p* < 0.001, d = 1.03; NRS-2002: 3.23 ± 0.77 [95% CI: 2.96–3.50] at T1 vs. 2.60 ± 0.65 [95% CI: 2.37–2.83] at T4, *p* < 0.001, d = 0.89; BMI: 24.01 ± 3.52 kg/m^2^ [95% CI: 22.78–25.24] at T1 vs. 24.87 ± 3.58 kg/m^2^ [95% CI: 23.62–26.12] at T4, *p* = 0.01, d = 0.24). However, analysis of quality of life assessed with the FAACT questionnaire did not demonstrate statistically significant differences between IG and CG (*p* > 0.05)—see Section 4.2. Nevertheless, correlation analysis showed that quality of life was significantly associated with nutritional status, functional performance, and appetite, detailed correlation results are presented in Section 4.6.

Analysis of QoL according to BMI categories showed no statistically significant differences between patient groups at T1 and T4 (Table 6 and Table 7), with small to medium effect sizes (η^2^ ranging from 0.01 to 0.10 across subscales). The lowest values for PWB, SWB, EWB, FWB, ACS, FACT-G, TOI, and FAACT were observed in patients with a BMI ≤ 18.9, whereas the highest values were found in patients with normal weight and overweight. However, these differences did not reach statistical significance (*p* > 0.05; Table 6 and Table 7). In patients with BMI ≥ 30, QoL values were intermediate and did not differ significantly from those in other BMI categories (*p* > 0.05). Although no statistically significant differences in QoL were observed across BMI categories at baseline, effect size estimates indicated potentially clinically relevant effects for social well-being (η^2^ = 0.10), functional well-being (η^2^ = 0.07), and FACT-G (η^2^ = 0.07), suggesting an association between BMI and selected QoL domains.

### 4.6. The Association Between Quality of Life and Functional Performance, Nutritional Status and Appetite

#### 4.6.1. Baseline (T1)—Overall Group

In baseline (T1) correlation analyses across for the entire cohort, significant associations were observed between QoL assessed with FAACT and selected indicators of nutritional status, functional performance, and appetite.

**Quality of life (FAACT, FACT-G, TOI, subscales)**

- The global FAACT score correlated positively with better nutritional status assessed by SGA (ρ = 0.25, 95% CI: 0.02–0.46, *p* = 0.03), higher functional performance (Karnofsky, ρ = 0.33, 95% CI: 0.11–0.52, *p* = 0.01), and better appetite (VAS, ρ = 0.49, 95% CI: 0.29–0.65, *p* < 0.001).
- The FACT-G score was significantly associated with SGA (ρ = 0.24, 95% CI: 0.01–0.45, *p* = 0.04), functional performance (Karnofsky, ρ = 0.24, 95% CI: 0.01–0.45, *p* = 0.04), and appetite (VAS, ρ = 0.38, 95% CI: 0.16–0.56, *p* < 0.001).
- TOI correlated positively with SGA (ρ = 0.25, 95% CI: 0.02–0.46, *p* = 0.03), Karnofsky (ρ = 0.31, 95% CI: 0.08–0.51, *p* = 0.01), and appetite (VAS, ρ = 0.49, 95% CI: 0.29–0.65, *p* < 0.001).

**Subscale-level results**

- The ACS (anorexia–cachexia symptoms) was higher in patients with better nutritional status (SGA, ρ = 0.23, 95% CI: 0.00–0.44, *p* = 0.049), greater functional performance (Karnofsky, ρ = 0.39, 95% CI: 0.17–0.57, *p* < 0.001), and VAS (ρ = 0.59, 95% CI: 0.42–0.72, *p* < 0.001).
- Physical Well-Being (PWB) correlated positively with appetite (VAS, ρ = 0.36, 95% CI: 0.14–0.55, *p* < 0.001) but negatively with NRS2002 (ρ = –0.28, 95% CI: −0.48–−0.05, *p* = 0.02), suggesting that malnutrition was associated with poorer physical functioning.
- Emotional Well-Being (EWB) correlated with functional performance (Karnofsky, ρ = 0.30, 95% CI: 0.07–0.50, *p* = 0.01) and appetite (VAS, ρ = 0.40, 95% CI: 0.19–0.58, *p* < 0.001).
- Functional Well-Being (FWB) correlated positively with appetite (VAS, ρ = 0.28, 95% CI: 0.05–0.48, *p* = 0.01).
- Social/Family Well-Being (SWB) correlated positively with appetite (VAS, ρ = 0.27, 95% CI: 0.04–0.47, *p* = 0.02).

**Inter-subscale FAACT correlations (T1)**

Analysis showed significant positive associations across all QoL domains. Strong correlations were observed between PWB and ACS (ρ = 0.66, 95% CI: 0.51–0.77, *p* < 0.001), and between EWB and ACS (ρ = 0.66, 95% CI: 0.51–0.77, *p* < 0.001). Additional strong associations included EWB with PWB (ρ = 0.59, 95% CI: 0.42–0.72, *p* < 0.001) and EWB with FWB (ρ = 0.57, 95% CI: 0.39–0.71, *p* < 0.001). Other correlations, though lower, remained moderate (e.g., PWB and FWB, ρ = 0.42, 95% CI: 0.21–0.59, *p* < 0.001; FWB and ACS, ρ = 0.44, 95% CI: 0.23–0.61, *p* < 0.001).

These findings indicate co-occurrence of positive changes across different QoL domains, suggesting that improvement in one area, such as physical well-being, is significantly linked with improvement in emotional, functional, and cachexia-related aspects.

**CG vs. IG correlations**

Correlation analyses performed separately for CG and IG revealed similar patterns—both functional performance and appetite correlated positively with QoL. As no significant differences were found between groups, detailed results are not presented in the main text.

#### 4.6.2. End of Observation (T4)—Overall Group

After 12 weeks of observation (T4), analysis confirmed that QoL remained significantly associated with nutritional status, functional performance, and appetite. All identified correlations were statistically significant.

**Quality of life (FAACT, FACT-G, TOI, subscales)**

At T4, the global FAACT score correlated positively with better nutritional status (SGA, ρ = 0.34, 95% CI: 0.12–0.53, *p* = 0.01) and greater appetite (VAS, ρ = 0.36, 95% CI: 0.14–0.55, *p* = 0.01).

In contrast to baseline, no significant correlation was observed between FAACT and functional performance (Karnofsky). Compared with T1, the association with SGA became slightly stronger, whereas the correlation with appetite was somewhat weaker, though still statistically significant.

A similar pattern was observed for FACT-G, which at T4 correlated with nutritional status (SGA, ρ = 0.32, 95% CI: 0.10–0.51) and appetite (VAS, ρ = 0.29, 95% CI: 0.06–0.49). Compared with T1, the correlation with SGA strengthened, while the earlier association with Karnofsky performance status was no longer significant.

The TOI score showed positive correlations with SGA (ρ = 0.38, 95% CI: 0.16–0.56), Karnofsky (ρ = 0.27, 95% CI: 0.04–0.47), and VAS (ρ = 0.41, 95% CI: 0.20–0.59). Relative to T1, the correlation with SGA increased, whereas those with functional performance and appetite slightly decreased in strength.

**Subscale-level results**

At the subscale level, the correlation patterns remained largely similar to those observed at T1, with some differences in strength and scope.

The ACS (Anorexia–Cachexia Symptoms) subscale remained significantly correlated with better nutritional status (SGA, ρ = 0.36, 95% CI: 0.14–0.55, *p* < 0.001), higher functional performance (Karnofsky, ρ = 0.37, 95% CI: 0.15–0.55, *p* < 0.001), and greater appetite (VAS, ρ = 0.46, 95% CI: 0.26–0.63, *p* < 0.001). Additionally, a new significant association emerged with nutritional risk (NRS2002, ρ = 0.26, 95% CI: 0.03–0.46, *p* = 0.04), which was not observed at baseline.

At T1, the PWB (Physical Well-Being) subscale correlated positively with appetite (VAS, ρ = 0.28, 95% CI: 0.05–0.48, *p* = 0.03). However, the strength of this association was weaker compared with baseline, and the previously observed negative correlation with NRS2002 was no longer significant.

EWB (Emotional Well-Being) at T4 remained significantly associated only with functional performance (Karnofsky, ρ = 0.32, 95% CI: 0.10–0.51, *p* = 0.02), while its earlier correlation with appetite (VAS) observed at T1 disappeared.

The FWB (Functional Well-Being) subscale showed a stronger correlation with appetite (VAS, ρ = 0.39, 95% CI: 0.17–0.57, *p* < 0.001 vs. 0.28 at T1), whereas the SWB (Social/Family Well-Being) subscale maintained a similar level of association with appetite (VAS, ρ = 0.29, 95% CI: 0.06–0.49, *p* = 0.03).

Additionally, at T4, a moderate positive correlation was observed between appetite (VAS) and nutritional status (SGA, ρ = 0.42, 95% CI: 0.21–0.59, *p* < 0.001), indicating that patients in better nutritional condition also reported higher appetite. This association was not significant at baseline.

**Inter-subscale FAACT correlations (T4)**

Analysis of internal associations among FAACT subscales at T4 revealed sustained positive and statistically significant correlations across all QoL domains. Compared with T1, several correlations strengthened notably, particularly between PWB and EWB (ρ = 0.71, 95% CI: 0.57–0.81, *p* < 0.001 vs. 0.59 at T1), EWB and FWB (ρ = 0.68, 95% CI: 0.53–0.79, *p* < 0.001 vs. 0.57), and PWB and FWB (ρ = 0.63, 95% CI: 0.47–0.75, *p* < 0.001 vs. 0.42). The correlations between EWB and ACS (ρ = 0.65, 95% CI: 0.49–0.77, *p* < 0.001) and between PWB and ACS (ρ = 0.65, 95% CI: 0.49–0.77, *p* < 0.001) remained at a similar level to that observed at baseline.

**CG vs. IG correlations**

Separate analyses performed at T4 confirmed that nutritional status, appetite, and functional performance remained significantly associated with QoL in both the control group (CG) and the intervention group (IG). Compared with baseline (T1), the overall pattern of associations was largely consistent, indicating that the relationship between QoL and key clinical parameters persisted over time. However, at T4 a broader range of correlations was noted in the CG and slightly fewer in the IG, suggesting some variability in the strength and number of associations between groups. Despite these minor differences, no statistically significant between-group differences were detected; therefore, detailed results are not presented in the main text.

## 5. Discussion

Assessment of quality of life (QoL) in patients with colorectal cancer (CRC) is an essential aspect of treatment. Tools such as the FAACT, FACT-G, and Karnofsky Performance Status (KPS) scales allow for the evaluation of changes across multiple dimensions of functioning [45]. Although current evidence on QoL in CRC patients using the FAACT is limited, the available studies provide a basis for placing our results in a broader clinical context.

**Performance Status**

In our study, mean Karnofsky Performance Status scores remained stable throughout the 12-week observation period in both the IG and the control group CG, with no statistically significant changes over time (*p* > 0.05) and no differences between groups (*p* > 0.05). Effect size estimates for changes in KPS were small (Kendall’s W ≤ 0.08; rank-biserial correlation r ≤ 0.23), indicating a negligible magnitude of change over the observation period. These findings indicate that ONS did not significantly affect overall physical performance in CRC patients with asymptomatic pre-cachexia. Still, it contributed to maintaining stability of functional status during chemotherapy.

Consistent with correlation analyses, no significant relationship between global FAACT scores and Karnofsky Performance Status was observed at 12 weeks, indicating that functional stability was maintained rather than improved.

The small effect sizes observed for these relationships suggest that changes in subjective quality of life were largely independent of objective performance status in this cohort.

A stable level of performance during active oncological treatment may be interpreted as a favorable outcome, since many studies report gradual decreases in KPS during systemic therapy, particularly in patients with cachexia [46,47]. Fearon et al. (2003) [48] showed that in patients with gastrointestinal cancer, supplementation with a protein- and n-3 fatty acid-enriched formula reduced muscle mass loss and maintained stable performance status (KPS), although no significant between-group differences were observed in KPS or QoL. In our cohort, the absence of deterioration in KPS suggests that early nutritional intervention—even in the absence of significant improvements in mean FAACT scores—may help preserve functional abilities. This observation is supported by the findings of Orsso et al. (2024) [49], who demonstrated that high-protein supplements, including those enriched with omega-3 fatty acids, improved performance status in some oncology patients. Furthermore, a meta-analysis by Dan X. et al. (2024) [50] summarized evidence on nutritional management in chemotherapy patients, highlighting KPS as a valuable indicator of functional performance in this population.

**Nutritional Status**

Our results demonstrate an association between nutritional status and subjective QoL in CRC patients with asymptomatic pre-cachexia. In IG, where ONS was implemented, significant improvements were noted in nutritional parameters (SGA, NRS-2002, BMI), while FAACT scores remained stable during 12 weeks of chemotherapy. Effect size estimates for changes in nutritional status indicated moderate to large effects (Cohen’s d ranging from 0.89 to 1.03), supporting the clinical relevance of the observed improvements despite stable mean QoL scores. Although mean FAACT values did not reach statistical significance at the group level, MID analysis showed that a substantial proportion of patients experienced clinically meaningful improvements, particularly in the ACS related to anorexia/cachexia symptoms (*n* = 16; 22% of patients). Between-group comparisons indicated medium to large effect sizes for global quality of life, physical well-being, and emotional well-being, suggesting that clinically relevant improvement was more likely in the intervention group than in the control group.

Analysis of QoL by BMI category showed no statistically significant differences. The lowest QoL scores were observed in patients with BMI ≤18.9, and the highest in those with normal weight and overweight, though differences were nonsignificant. However, effect size estimates for selected QoL domains ranged from small to medium (η^2^ = 0.01–0.10), with medium effects observed for social well-being, functional well-being, and FACT-G, suggesting potentially clinically relevant associations between BMI and specific QoL dimensions. This supports the view that BMI alone is not a sufficient predictor of QoL in oncology; functional performance, appetite, and overall nutritional status play more critical roles.

FAACT and its subscales, including ACS, TOI, and FACT-G, correlated significantly with better nutritional status as assessed by SGA, at baseline and at 12 weeks. The strength of these associations was small, suggesting a weak but consistent relationship between better nutritional status and higher quality-of-life scores in selected domains. Notably, Physical Well-Being (PWB), Functional Well-Being (FWB) and Anorexia/Cachexia Subscale (ACS) scores also correlated with higher appetite (VAS) (strength of these associations was small to moderate), underscoring the importance of subjective hunger as a predictor of QoL decline. These findings are consistent with Blauwhoff-Buskermolen et al. (2016), who reported that cancer patients with lower ACS scores (≤37 points) had poorer appetite, suggesting this cut-off for identifying anorexia [51]. Furthermore, Abraham et al. (2019) [52] showed that in patients with esophageal/gastric cancers, ACS >37 points was associated with more prolonged overall survival compared to ACS ≤ 37 (median OS: 19.3 vs. 6.7 months, HR 2.9, 95% CI 1.4–6.0; *p* < 0.0001). Notably, the FAACT ACS scale was a stronger predictor of OS in metastatic patients than BMI or 6-month weight loss [52]. These insights may be relevant for long-term survival in our studied cohort.

**FAACT**

In our trial, 12-week ONS did not result in statistically significant changes in total FAACT (*p* = 0.09), FACT-G (*p* = 0.22), or TOI (*p* = 0.17) compared with CG, which received only dietary counseling.

However, the observed change in total FAACT corresponded to a medium effect size (r = 0.30), suggesting a potentially meaningful magnitude of change despite the lack of statistical significance at the group level.

Notably, MID analysis revealed that many patients—13 for FAACT and 14 for FACT-G—achieved improvements ≥ MID thresholds (11.41 and 8.35 points, respectively). Improvements were observed in ACS (*n* = 16) and EWB (*n* = 14). Furthermore, between-group comparisons showed significantly higher proportions of clinically relevant improvement in IG versus CG for global QoL (FAACT), physical well-being (PWB), and especially emotional well-being (EWB). These between-group differences were accompanied by medium to large effect sizes (odds ratios ≥ 5.50; φ up to 0.40), indicating that clinically meaningful improvement was substantially more likely in the intervention group than in the control group. These findings suggest that ONS may not only stabilize nutritional status but also translate into clinically meaningful improvements in selected key QoL dimensions for individual patients, even when mean group-level changes are not statistically significant.

Additionally, improvements in single items were noted: satisfaction with coping with illness (GE2, *p* = 0.003) and subjective perception of health improvement (ACT13, *p* < 0.05). These item-level improvements indicate enhanced emotional adaptation and illness perception, highlighting the sensitivity of single-item FAACT analysis to detect patient-level benefits.

Similarly, Tan et al. (2021) observed no group-level improvement in QoL after ONS in postoperative CRC patients, but they reported increases in muscle mass and fewer treatment modifications (delays, dose reductions, treatment interruptions) compared with dietary counseling alone [53].

Meta-analyses further confirm the effectiveness of nutritional interventions. Baldwin et al. (2012) found that in malnourished cancer patients, nutritional support improved intake and certain QoL domains (emotional functioning, dyspnea, appetite loss, overall QoL), though without mortality benefit (RΡ = 1.06, 95% CI: 0.92–1.22, *p* = 0.43) [54]. In a systematic review and meta-analysis, ONS significantly increased body weight and improved nutritional status, while its effect on global QoL was borderline significant (MD +4.01, 95% CI: 0.08–7.94; *p* = 0.05). However, significant reductions in fatigue were noted (MD −7.63, 95% CI: −13.87 to −1.39; *p* = 0.02) [55]. Habibi et al. also reported that ONS significantly improved weight gain, nutritional status, reduced fatigue, and enhanced QoL, with the greatest benefits observed at doses ≥500 mL/day [56].

**Interpretation of MID versus Mean Analysis**

Although MID analysis demonstrated clinically meaningful improvement in a proportion of patients receiving ONS, statistically significant differences in mean FAACT, FACT-G, and TOI scores between groups were not observed over 12 weeks. This discrepancy reflects the fundamental distinction between clinical and statistical interpretation of QoL outcomes. Notably, the upward trend in mean FAACT scores in the intervention group was accompanied by a medium effect size (r = 0.30), suggesting that the absence of statistical significance may be related to limited statistical power rather than the lack of a true intervention effect. MID assesses meaningful change at the individual level and therefore identifies patients who benefit despite overall group stability. In contrast, mean-based analysis averages scores across heterogeneous disease stages, treatment toxicity, psychological state, and nutritional phenotype, which can dilute individual changes and lead to a nonsignificant group result.

These findings suggest that while ONS may not alter mean HRQoL outcomes at the population level, it may benefit selected patients who are responsive to nutritional support—particularly in domains related to emotional and physical well-being. Such differentiation is essential for clinical practice, indicating that nutritional intervention should be individualized rather than universally expected to improve global QoL scores in CRC patients undergoing chemotherapy.

**FAACT Subscales**

In our study, no significant changes were observed in most FAACT items (PWB, FWB, SWB, EWB, and ACS). These analyses were accompanied by very small effect sizes (Kendall’s W typically ≤ 0.05), indicating true stability of the assessed QoL domains during the 12-week observation period rather than a lack of sensitivity of the measurement tools. The results suggest relative stability in somatic symptoms (pain, nausea, general well-being), ability to work, sleep quality, and social support during the 12-week observation period. This stability may reflect the chronic nature of CRC-related burdens and patient adaptation and is consistent with the small magnitude of observed effects.

Similar patterns have been reported in other cancers. Hwang et al. found that in advanced cancer patients, FACT-G SWB scores remained stable, while PWB and EWB declined only in the last 2–3 months of life [57]. Likewise, in breast cancer patients undergoing radiotherapy, FACT-B scores remained stable during short-term follow up, highlighting the persistence of physical and functional components [58].

Our study, unlike most CRC research, analyzed results at the single-item FAACT level. Prior CRC studies typically reported only global or subscale scores [59]. Single-item analyses were previously conducted mainly in validation studies. For example, Ribaudo et al. (2000) [60] revalidated the ACS, identifying limited value in items such as “I have diarrhea” or “I have nausea.”

In our cohort, meaningful improvements were noted in GE2 (“I am satisfied with how I am coping with my illness”) and ACT13 (“My overall condition is improving”), suggesting that CRC patients may adapt emotionally over time, despite persistent cachexia-related symptoms. These improvements occurred in domains related to emotional adaptation and illness perception and are consistent with the moderate effect size observed for total FAACT in the intervention group (r = 0.30) as well as with MID-based analyses showing clinically meaningful benefits in emotional well-being. Near-significant trends in GS4 and GS5 (acceptance and communication within family) may reflect gradual increases in perceived social support. The lack of changes in most ACS items indicates that cachexia symptoms (loss of appetite, vomiting, early satiety) remained persistent.

Notably, the lowest scores were observed in EWB at both baseline and week 12, suggesting that the emotional domain is particularly vulnerable during systemic treatment, even in pre-cachectic states. Ximenes et al. (2020) [61] reported similarly poor emotional and physical scores at diagnosis in elderly oncology patients, though they observed improvements in emotional functioning over 6–12 months, possibly due to holistic care. Our shorter 12-week period and intensive systemic treatment may explain differences. In contrast, Ebansen et al. found no improvement in emotional functioning at 3 and 6 months post-diagnosis [62].

In our cohort, SWB had the highest scores, consistent with Sharour et al., who found social/family well-being scored highest (mean 23.4, SD 3.8) and emotional well-being lowest (mean 16.2, SD 4.5) [59].

Analysis by sex showed no significant differences in QoL. Laghousi et al., however, found women reported worse physical and social functioning, greater fatigue, and pain than men [63]. Zhou et al. concluded that female patients tend to experience more psychological distress and complex social functioning, but similarities in coping and psychosocial outcomes are also present [64]. The absence of gender-related differences in our cohort may reflect comparable baseline nutritional and functional status and early treatment stage.

Although significant improvements in nutritional status were observed in the intervention group, these changes did not translate into statistically significant improvements in quality of life at the group level. This finding is consistent with the very small effect sizes observed for most FAACT subscales and the selective presence of moderate effects (e.g., total FAACT in IG, r = 0.30) and medium-to-large effects in MID-based analyses. Several factors may explain this discrepancy. First, the 12-week follow-up period may have been insufficient to capture broader QoL changes, particularly in domains related to emotional and functional well-being, which often require longer timeframes to improve. Second, the relatively high proportion of patients with advanced disease may have limited the responsiveness of QoL outcomes, as disease burden and treatment-related symptoms can outweigh the potential benefits of nutritional improvement. Finally, quality of life is a multidimensional construct influenced by nutritional status and psychological, social, and treatment-related factors. Therefore, improvements in objective nutritional parameters may not be accompanied by parallel changes in patients’ subjective well-being over a short observation period.

**Potential Confounding Variables**

Several clinical factors may have influenced quality-of-life outcomes independently of oral nutritional supplementation and should be considered when interpreting the results of this study.

A substantial proportion of the study population presented with advanced disease, including stage III–IV colorectal cancer and metastatic disease. Advanced tumor burden and cancer-related symptoms are known to exert a substantial negative impact on quality of life. They May limit the potential for measurable improvement over a relatively short follow-up period. Consequently, the presence of advanced disease may have attenuated the observable effects of nutritional intervention on group-level QoL outcomes.

In addition, patients in the study received different chemotherapy regimens, including FOLFOX, FOLFIRI, and XELOX. These regimens differ in their toxicity profiles, with FOLFIRI being associated with greater gastrointestinal toxicity. Treatment-related adverse effects such as nausea, diarrhea, fatigue, and appetite loss may have influenced physical and functional well-being independently of nutritional support, potentially confounding QoL assessments.

The presence of a colostomy in some patients is another relevant factor. Colostomy care can affect emotional well-being, body image, functional independence, and social functioning. These aspects of quality of life may not be directly modified by nutritional interventions alone and could therefore contribute to the lack of statistically significant improvements in certain QoL domains.

Finally, baseline differences in nutritional status between the intervention and control groups may have influenced individual responsiveness to nutritional support. Patients with poorer baseline nutritional status may be more likely to experience clinically meaningful improvements following supplementation, which could partly explain the higher proportion of responders observed in the intervention group. This phenomenon may also reflect regression to the mean and interindividual variability in response to nutritional interventions.

Taken together, these factors highlight the multifactorial nature of quality of life in patients with colorectal cancer and suggest that nutritional intervention alone may be insufficient to overcome the combined impact of advanced disease, treatment-related toxicity, and psychosocial burden during active chemotherapy.

The current results should be interpreted with caution. The absence of statistically significant group-level improvements does not negate the clinical value of oral nutritional supplementation (ONS) for individual patients. Rather, it highlights the heterogeneity of quality-of-life responses, which may be more accurately captured by minimally important difference (MID) analyses than by comparisons of aggregated mean scores. Importantly, the presence of moderate effect sizes in selected quality-of-life domains, despite the lack of statistical significance, suggests a potentially clinically meaningful impact of the intervention that may reach statistical significance with a larger sample size or longer follow-up period. Consequently, early ONS may be particularly beneficial for specific patient subgroups, such as those with anorexia/cachexia symptoms, emotional distress, or impaired physical functioning, rather than for the entire colorectal cancer population.

**Limitations**

This study involved multiple statistical comparisons, including analyses of several FAACT subscales, individual questionnaire items, and subgroup analyses. As a result, there is an increased risk of type I error. Therefore, analyses conducted at the level of individual questionnaire items should be interpreted as exploratory. Future studies with larger sample sizes and prespecified hypotheses are warranted to confirm these findings.

Importantly, the absence of statistically significant effects in some analyses should not be interpreted as evidence of a lack of association or intervention effect. Nonsignificant results may reflect several factors, including a true absence of effect in the studied sample, an insufficient duration of follow-up to capture changes over time, or limited statistical power to detect moderate effects. Consequently, the risk of type II error cannot be excluded, particularly in analyses of quality-of-life outcomes characterized by substantial interindividual variability.

Although effect size estimates are independent of sample size, statistical significance is strongly influenced by it. Therefore, clinically meaningful effects—especially those of moderate magnitude—may fail to reach statistical significance in studies with relatively small sample sizes or short observation periods. Validation of the observed effect sizes in larger cohorts and with longer follow-up is necessary to determine their robustness and clinical relevance.

**Selection Bias and Missing Data**

Selection bias should be considered when interpreting the results of this study. Only patients who completed all quality-of-life questionnaires at each assessment time point were included in the final analyses. As a result, patients who experienced clinical deterioration, hospitalization, treatment discontinuation, or symptom burden severe enough to prevent questionnaire completion may be underrepresented. This approach, while commonly applied in longitudinal QoL studies, may have led to a study sample consisting of relatively fitter and more adherent patients, potentially limiting the generalizability of the findings.

Complete-case analysis was chosen to ensure consistency and reliability of longitudinal comparisons; however, this approach may have further reduced the effective sample size and increased the risk of type II error. Future studies with larger populations, extended follow-up periods, and advanced methods for handling missing data are needed to better characterize the impact of nutritional interventions on quality of life across diverse patient subgroups.

## 6. Conclusions

The results indicate that in colorectal cancer patients with pre-cachexia, oral nutritional supplementation (ONS) maintained stable performance status and did not result in statistically significant changes in mean quality-of-life (QoL) scores at the group level over a 12-week period. Emotional well-being was the most impaired QoL domain, whereas social/family well-being remained the strongest. Although no statistically significant differences were observed between the intervention and control groups, ONS increased the proportion of patients achieving clinically meaningful improvement, particularly in the physical and emotional domains. This pattern, supported by small-to-moderate effect sizes despite the absence of group-level statistical significance, suggests a clinically relevant benefit for selected patients who are responsive to nutritional support.

Regular monitoring of appetite and cachexia-related symptoms may facilitate timely, individualized nutritional interventions with potential prognostic benefits. Given the relatively short follow-up period and modest sample size, these findings should be interpreted with caution, as the absence of statistical significance may partly reflect limited statistical power and the risk of type II error. Nevertheless, item-level FAACT analyses identified domains most sensitive to change, providing a rationale for more personalized nutritional and psycho-oncological strategies. Larger studies with longer follow-up across different stages of cachexia are warranted to validate the observed effect sizes and to further refine supportive care interventions in this population.

## Figures and Tables

**Table 1:** Baseline demographic and clinical characteristics of the intervention (IG) and control (CG) groups.

Variable	IG (*N* = 40)	CG (*N* = 32)	*p*-Value
Age (years), mean ± SD	72.62 ± 10.56	71.28 ± 11.68	0.639
Sex, female/male, *n*	17/23	19/13	0.155
Tumor grade, *n* (%)	G2: 34 (85%) G3: 4 (10%) G4: 2 (5%)	G2: 29 (90.6%) G3: 3 (9.4%) G4: 0 (0%)	0.305
Metastases, *n* (%)	34 (85%)	31 (97%)	0.096
Colostomy, *n* (%)	21 (52.5%)	6 (18.75%)	0.003
Chemotherapy regimen, *n* (%)	FOLFOX: 14 (35%) FOLFIRI: 4 (10%) Other: 22 (55%)	FOLFOX: 10 (31.3%) FOLFIRI: 5 (15.6%) Other: 17 (53.1%)	0.765

Mann–Whitney U test (age); Pearson χ^2^ test (sex, chemotherapy); Fisher–Freeman–Halton exact test (tumor grade); Fisher’s exact test (metastases, colostomy).

**Table 2:** MID—All patients.

	SD T1	MID 0.5 × SD	MID+ * (*n*)	MID− ** (*n*)
FAACT	22.82	11.41	13	8
FACT-G	16.70	8.35	14	8
PWB	6.44	3.22	13	10
SWB	4.03	2.01	10	11
EWB	5.15	2.57	14	8
FWB	6.10	3.05	12	4
ACS	7.89	3.95	16	6

* MID+ (improvement—clinically meaningful change perceived by the patient). ** MID− (deterioration—clinically meaningful change perceived by the patient).

**Table 3:** Comparison of MID+ and MID−: IG vs. CG.

Scale	Outcome	CG (*n* = 25)	IG (*n* = 34)	*p*-Value	OR	95% CI	φ	Interpretation
FAACT	Improvement	2/25	11/34	0.03	5.5	[1.1, 27.62]	0.29	Improvement is significantly more common in IG
FAACT	Deterioration	3/25	5/34	1.0	1.26	[0.27, 5.87]	0.04	
FACT-G	Improvement	5/25	9/34	0.76	1.44	[0.42, 4.98]	0.08	No significant differences
FACT-G	Deterioration	4/25	4/34	0.71	0.7	[0.16, 3.12]	0.06	
PWB	Improvement	2/25	11/34	0.03	5.5	[1.1, 27.62]	0.29	Improvement is significantly more common in IG
PWB	Deterioration	6/25	4/34	0.30	0.42	[0.11, 1.69]	0.16	
SWB	Improvement	5/25	5/34	0.73	0.69	[0.18, 2.7]	0.07	No significant differences
SWB	Deterioration	6/25	5/34	0.50	0.55	[0.15, 2.04]	0.12	
EWB	Improvement	1/25	13/34	0.002	14.86	[1.79, 123.36]	0.40	Improvement is significantly more common in IG
EWB	Deterioration	5/25	3/34	0.26	0.39	[0.08, 1.8]	0.16	
FWB	Improvement	2/25	10/34	0.05	4.79	[0.95, 24.27]	0.26	No significant differences
FWB	Deterioration	2/25	2/34	1.00	0.72	[0.09, 5.48]	0.04	
ACS	Improvement	6/25	10/34	0.77	1.32	[0.41, 4.28]	0.06	No significant differences
ACS	Deterioration	5/25	1/34	0.07	0.12	[0.01, 1.11]	0.28	

**Table 4:** Comparison of Karnofsky Performance Status results over 12 weeks (percentage of patients).

KPS Overall Group	T1	T2	T3	T4
70	1 (1.4%, 0.2–7.5%)	1 (1.5%, 0.3–8.0%)	1 (1.7%, 0.3–8.9%)	0 (0.0%, 0.0–6.1%)
80	8 (11.1%, 5.7–20.4%)	7 (10.4%, 5.2–20.0%)	8 (13.3%, 6.9–24.2%)	8 (13.6%, 7.0–24.5%)
90	38 (52.8%, 41.4–63.9%)	36 (53.7%, 41.9–65.1%)	30 (50.0%, 37.7–62.3%)	31 (52.5%, 40.0–64.7%)
100	25 (34.7%, 24.8–46.2%)	23 (34.3%, 24.1–46.3%)	21 (35.0%, 24.2–47.6%)	20 (33.9%, 23.1–46.6%)

Note: Values are presented as *n* (%, 95% CI).

**Table 5:** Comparison of Karnofsky Performance Status results over 12 weeks (overall group, IG, CG).

Group	T1	T2	T3	T4	*p*-Value ^1^	W
Overall	92.08 ± 6.91	92.09 ± 6.86	91.67 ± 7.85	92.04 ± 6.64	0.37	0.02
	(90.25–93.91)	(90.27–93.91)	(89.59–93.75)	(90.28–93.80)		
	90 (70–100)	90 (70–100)	90 (60–100)	90 (80–100)		
CG	93.75 ± 6.09	94.14 ± 5.68	93.60 ± 5.68	92.50 ± 6.76	0.12	0.08
	(91.24–96.26)	(91.80–96.48)	(91.26–95.94)	(89.71–95.29)		
	90 (80–100)	90 (80–100)	90 (80–100)	90 (80–100)		
IG	90.75 ± 7.30	90.53 ± 7.33	90.29 ± 8.57	91.71 ± 6.64	0.52	0.02
	(88.12–93.38)	(87.89–93.17)	(87.20–93.38)	(89.32–94.10)		
	90 (70–100)	90 (70–100)	90 (70–100)	90 (80–100)		
*p*-value ^2^	0.08	0.13	0.13	0.65		
r	0.23	0.2	0.2	0.06		
(CG vs. IG)						

Notes: ^1^ *p*-value for changes over time (Friedman test); W = Kendall’s coefficient of concordance (effect size). ^2^ *p*-value for comparison between CG and IG at each time point (Mann–Whitney U test); r = rank-biserial correlation (effect size). Sample sizes: Overall group (*n* = 57), CG (*n* = 25), IG (*n* = 32).

**Table 6:** Quality of Life (FAACT) According to BMI Categories—T1.

BMI Category	*n*	PWB	SWB	EWB	FWB	ACS	FACT-G	TOI	FAACT
≤18.9	7	13.43 ± 5.68 CI: 8.18–18.68 15.00 (5.00–20.00)	18.86 ± 3.08 CI: 16.01–21.71 18.00 (14.00–24.00)	13.14 ± 2.73 CI: 10.62–15.66 12.00 (10.00–18.00)	14.86 ± 4.38 CI: 10.81–18.91 14.00 (9.00–21.00)	26.43 ± 6.02 CI: 20.86–32.00 25.00 (19.00–36.00)	60.29 ± 10.44 CI: 50.63–69.95 57.00 (45.00–75.00)	54.71 ± 14.23 CI: 41.55–67.87 52.00 (37.00–76.00)	86.71 ± 15.61 CI: 72.27–101.15 81.00 (70.00–109.00)
>18.9–24.9	31	19.23 ± 5.73 CI: 17.13–21.33 20.00 (6.00–28.00)	20.48 ± 4.16 CI: 18.95–22.01 21.00 (9.00–28.00)	14.97 ± 4.81 CI: 13.21–16.73 16.00 (3.00–24.00)	16.68 ± 6.14 CI: 14.43–18.93 16.00 (0.00–28.00)	31.10 ± 6.79 CI: 28.61–33.59 31.00 (17.00–44.00)	71.35 ± 15.91 CI: 65.51–77.19 71.00 (29.00–108.00)	67.00 ± 14.92 CI: 61.53–72.47 65.00 (41.00–100.00)	102.45 ± 20.67 CI: 94.87–110.03 100.00 (56.00–152.00)
≥25–29.9	24	17.92 ± 7.48 CI: 14.76–21.08 19.00 (5.00–28.00)	21.33 ± 4.35 CI: 19.49–23.17 23.50 (11.00–28.00)	13.25 ± 6.44 CI: 10.53–15.97 13.00 (0.00–24.00)	17.12 ± 6.96 CI: 14.18–20.06 16.50 (1.00–28.00)	32.83 ± 9.05 CI: 29.01–36.65 31.50 (15.00–48.00)	69.62 ± 19.58 CI: 61.35–77.89 67.00 (32.00–103.00)	67.88 ± 18.91 CI: 59.90–75.86 65.00 (39.00–104.00)	102.46 ± 26.89 CI: 91.11–113.81 100.00 (57.00–151.00)
≥30	10	17.70 ± 5.62 CI: 13.68–21.72 18.00 (8.00–24.00)	20.60 ± 3.41 CI: 18.16–23.04 19.50 (16.00–26.00)	16.20 ± 3.39 CI: 13.77–18.63 15.50 (11.00–21.00)	18.90 ± 4.84 CI: 15.44–22.36 19.50 (11.00–27.00)	29.80 ± 8.80 CI: 23.50–36.10 29.00 (14.00–43.00)	73.40 ± 14.58 CI: 62.97–83.83 72.50 (48.00–94.00)	66.40 ± 17.44 CI: 53.92–78.88 66.50 (33.00–94.00)	103.20 ± 22.24 CI: 87.29–119.11 102.00 (62.00–137.00)
*p*-value ^1^		0.34	0.08	0.35	0.32	0.69	0.19	0.83	0.64
η^2^		0.05	0.10	0.05	0.07	0.02	0.07	0.01	0.02

Notes: Mean ± SD; 95%CI; Median (Min–Max); ^1^ *p*-value from ANOVA Welch test comparing BMI categories. Sample size *N* = 72.

**Table 7:** Quality of Life (FAACT) According to BMI Categories—T4.

BMI Category	*n*	PWB	SWB	EWB	FWB	ACS	FACT-G	TOI	FAACT
≤18.9	3	16.33 ± 6.11 CI: −4.61–37.27 15.00 (11.00–23.00)	19.33 ± 4.16 CI: 8.86–29.80 18.00 (16.00–24.00)	12.67 ± 6.43 CI: −9.53–34.87 10.00 (8.00–20.00)	15.67 ± 4.73 CI: 3.37–27.97 14.00 (12.00–21.00)	24.00 ± 4.00 CI: 14.00–34.00 24.00 (20.00–28.00)	64.00 ± 21.38 CI: −5.74–133.74 57.00 (47.00–88.00)	56.00 ± 14.73 CI: 12.50–99.50 53.00 (43.00–72.00)	88.00 ± 25.24 CI: 6.30–169.70 81.00 (67.00–116.00)
>18.9–24.9	26	18.50 ± 6.10 CI: 16.04–20.96 19.50 (4.00–28.00)	20.23 ± 4.34 CI: 18.44–22.02 20.00 (10.00–28.00)	14.85 ± 4.24 CI: 13.14–16.56 15.00 (6.00–24.00)	18.42 ± 5.02 CI: 16.40–20.44 17.50 (8.00–28.00)	31.31 ± 7.46 CI: 28.30–34.32 32.50 (16.00–48.00)	72.00 ± 15.84 CI: 65.61–78.39 72.50 (28.00–107.00)	68.23 ± 15.73 CI: 61.88–74.58 70.50 (41.00–103.00)	103.31 ± 21.45 CI: 94.65–111.97 104.50 (57.00–155.00)
≥25–29.9	19	18.21 ± 6.91 CI: 14.88–21.54 18.00 (6.00–28.00)	21.16 ± 4.00 CI: 19.34–22.98 22.00 (14.00–28.00)	15.47 ± 5.21 CI: 12.96–17.98 15.00 (6.00–24.00)	19.16 ± 5.79 CI: 16.37–21.95 21.00 (6.00–28.00)	34.00 ± 5.93 CI: 31.14–36.86 32.00 (25.00–44.00)	74.00 ± 18.98 CI: 64.83–83.17 72.00 (38.00–104.00)	71.37 ± 16.66 CI: 63.33–79.41 72.00 (40.00–99.00)	108.00 ± 23.86 CI: 97.17–118.83 104.00 (66.00–147.00)
≥30	11	19.64 ± 6.39 CI: 15.35–23.93 19.00 (6.00–28.00)	18.82 ± 6.85 CI: 13.91–23.73 20.00 (1.00–28.00)	16.55 ± 4.03 CI: 13.85–19.25 16.00 (12.00–24.00)	20.18 ± 3.79 CI: 17.64–22.72 20.00 (15.00–28.00)	33.55 ± 5.26 CI: 30.02–37.08 32.00 (26.00–40.00)	75.18 ± 11.21 CI: 67.66–82.70 71.00 (64.00–103.00)	73.36 ± 13.44 CI: 64.33–82.39 71.00 (55.00–96.00)	108.73 ± 15.34 CI: 98.44–119.02 102.00 (91.00–143.00)
*p*-value ^1^		0.92	0.65	0.64	0.54	0.43	0.85	0.92	0.93
η^2^		0.01	0.02	0.02	0.03	0.04	0.01	0.01	0.01

Notes: Mean ± SD; 95% CI; Median (Min–Max); ^1^ *p*-value from ANOVA Welch test comparing BMI categories; Sample size *N* = 59.

## Data Availability

The data presented in this study are available on reasonable request from the corresponding author. The data are not publicly available due to privacy and ethical restrictions.

## Abbreviations

The following abbreviations are used in this manuscript:

ACS	Anorexia/Cachexia Subscale (FAACT Additional Concerns)
BMI	Body Mass Index
CG	Control Group
CI	Confidence Interval
CRC	Colorectal Cancer
ECOG	Eastern Cooperative Oncology Group (Performance Status)
EWB	Emotional Well-Being
FAACT	Functional Assessment of Anorexia/Cachexia Therapy
FACT-G	Functional Assessment of Cancer Therapy—General
FOLFOX-4	5-fluorouracil, leucovorin, and oxaliplatin regimen
FOLFIRI	5-fluorouracil, leucovorin, and irinotecan regimen
FWB	Functional Well-Being
HRQoL	Health-Related Quality of Life
IG	Intervention Group
KDOQI	Kidney Disease Outcomes Quality Initiative
KPS	Karnofsky Performance Status
MD	Mean Difference
MID	Minimally Important Difference
NRS-2002	Nutritional Risk Screening 2002
ONS	Oral Nutritional Supplements
OR	Odds Ratio
OS	Overall Survival
PWB	Physical Well-Being
QoL	Quality of Life
RR	Risk Ratio
SD	Standard Deviation
SGA	Subjective Global Assessment
SWB	Social/Family Well-Being
T1–T4	Study Time Points: baseline and weeks 4, 8, and 12
TNM (UICC)	Tumor–Node–Metastasis (Union for International Cancer Control)
TOI	Trial Outcome Index
VAS	Visual Analogue Scale
XELOX	Capecitabine and Oxaliplatin Regimen
